# Suppression of Railway Catenary Galloping Based on Structural Parameters’ Optimization

**DOI:** 10.3390/s24030976

**Published:** 2024-02-02

**Authors:** Yuhui Liu, Yang Song, Fuchuan Duan, Zhigang Liu

**Affiliations:** 1 School of Electrical Engineering, Southwest Jiaotong University, Chengdu 610031, China; liuyuhui0715@163.com (Y.L.); duanfc_cd@outlook.com (F.D.); liuzg_cd@126.com (Z.L.); 2 China State Railway Group Company, Ltd., Beijing 100089, China; 3 State Key Laboratory of Rail Transit Vehicle System, Southwest Jiaotong University, Chengdu 610031, China; 4 SWJTU-Leeds Joint School, Southwest Jiaotong University, Chengdu 610031, China

**Keywords:** railway, catenary, galloping, structure optimization, genetic algorithm

## Abstract

Railway catenary galloping, induced by aerodynamic instability, poses a significant threat by disrupting the electric current connection through sliding contact with the contact wire. This disruption leads to prolonged rail service interruptions and damage to the catenary’s suspension components. This paper delves into the exploration of optimizing the catenary system’s structure to alleviate galloping responses, addressing crucial parameters such as span length, stagger dropper distribution, and tension levels. Employing a finite element model, the study conducts simulations to analyze the dynamic response of catenary galloping, manipulating structural parameters within specified ranges. To ensure accurate and comprehensive exploration, the Sobol sequence is utilized to generate low-discrepancy, quasi-random, and super-uniform distribution sequences for the high-dimensional parameter inputs. Subsequent to the simulation phase, a genetic algorithm based on neural networks is employed to identify optimal parameter settings for suppressing catenary galloping, taking into account various constraints. The results gleaned from this investigation affirm that adjusting structural parameters can effectively diminish the galloping amplitude of the railway catenary. The most impactful strategy involves augmenting tension and reducing span length. Moreover, even when tension and span length are fixed, adjusting other parameters demonstrates efficacy in reducing galloping amplitudes. The adjustment of messenger-wire tension, dropper distribution, and stagger can achieve a 22.69% reduction in the maximum vertical galloping amplitude. Notably, maintaining a moderate stagger value and a short steady arm–dropper distance is recommended to achieve the minimum galloping amplitude. This research contributes valuable insights into the optimization of railway catenary systems, offering practical solutions to mitigate galloping-related challenges and enhance overall system reliability.

## 1. Introduction

**Figure 1:**
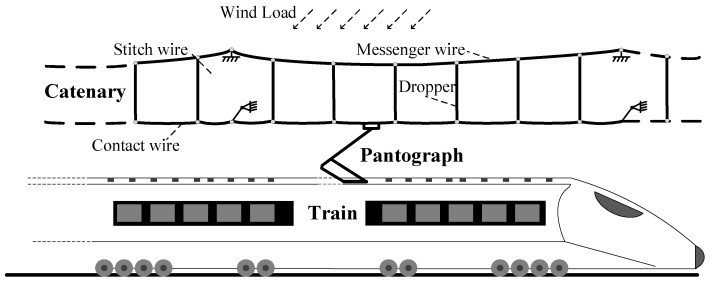
Schematics of pantograph-catenary system.

In electric railway systems, the catenary structure running alongside the tracks functions as a means to energize the electric train. Power is conveyed to the train by means of sliding contact with a pantograph affixed to the roof of the vehicle [1]. This arrangement, depicted in Figure 1, involves the catenary’s contact wire serving a dual role: facilitating the pantograph’s mechanical pathway and conducting the electrical current. The efficacy of the current collection predominantly hinges on the mechanical interaction between the contact wire and the pantograph collectors. However, this interaction quality is normally challenged by a number of environmental factors; among them, the wind load is one of the most significant due to the high flexibility and long-span structure of the catenary.

### 1.1. Problem Description

The impact of wind load on the catenary can be categorized into two distinct types. The initial one is referred to as buffeting, a form of forced vibration [2]. Elevated buffeting amplitude can lead to a decline in the quality of contact between the pantograph and the catenary [3]. The second type is self-induced vibration triggered by aerodynamic instability, known as galloping [4]. In comparison to buffeting, galloping boasts significantly greater vibration amplitude [5]. When galloping occurs, the pantograph’s ability to establish an electric current connection through sliding contact with the contact line is severely hindered, resulting in prolonged rail service disruptions. Moreover, galloping negatively impacts the catenary by causing damage to its components. Examples from China Railway Corporation depict the substantial galloping of the Luoyang-Xiangyang railway’s OCL in 2011, with an amplitude reaching 0.5 m, leading to hours of traffic disruption. Another instance occurred in 2003 on a segment of the Beijing-Guangzhou railway, causing considerable harm to catenary suspension components, including 31 steady arms, and 211 droppers fractured. These real-world instances underscore the substantial potential of catenary galloping to jeopardize the smooth operation of electric railways. Some previous works, like [6,7], have already pointed out that the galloping is caused by the abnormal profile of the contact wire, which may lead to aerodynamic instability. Currently, there are no effective measures to suppress galloping. Traditional measures, like including dampers and the optimization of contact-wire profiles are not likely to be implemented in catenary systems, as these measures may hinder the pantographs’ passage. The most economical and straightforward measure is to optimize the catenary’s structure in the design phase. Therefore, our understanding of the catenary structural parameters’ effect on galloping behavior should be clarified, and the optimization of catenary structural parameters deserves further discussion.

### 1.2. Literature Review

As a significant subdivision within railway dynamics [8,9], the examination of catenary dynamics primarily concentrates on the interaction between the pantograph and the catenary. This interaction holds substantial significance in diminishing pantograph arcing, enhancing dynamic performance [10], and ensuring effective current collection quality [11]. Employing field tests represents the most direct approach to replicating authentic pantograph–catenary interaction behaviors [12,13], though this approach often entails considerable financial expenses. Alternatively, the numerical model of the catenary proves to be the most efficient method for exploring catenary dynamics [14], refining structural parameters [15], and evaluating control strategies [16,17] to enhance interaction performance. However, its accuracy hinges on validation against measurement data. To encompass the intricate operational environment of the catenary system, the pantograph–catenary model aptly integrates vehicle-track perturbations [18]. To incorporate catenary errors into current collection quality assessments, appropriate models have been developed for contact-wire irregularities [19] and geometry deviations [20]. Their impacts on contact force are quantified through both deterministic [21] and stochastic analyses [22].

Regarding the influence of wind load, the preceding research predominantly focuses on catenary buffeting. For example, in [23,24], distinct approaches are employed to generate spatial wind fields from empirical wind speed spectra, subsequently deriving aerodynamic loads that act upon contact lines and affect the catenary model. An analysis of catenary buffeting responses ensues, alongside the quantification of their influence on the pantograph–catenary contact force. In [25], an aero-elastic model of the catenary undergoes wind tunnel testing, where buffeting responses are initially analyzed using experimental data. In [26], numerical simulations reproduce catenary buffeting, the accuracy of which is corroborated experimentally. As for catenary galloping, limited studies pertain to this subject matter. Only Scanlon et al. [6] construct a simplified single-degree-of-freedom catenary model and endeavor to decrease the likelihood of catenary galloping by applying mechanical damping. A parallel effort is replicated in [27] to explore the instability range of a contact line on a bridge. Song et al. [28] undertake a preliminary investigation of contact-line wear due to aerodynamics using computational fluid dynamics (CFD) and explore potential galloping amplitudes.

With the development of artificial intelligence, neural network and deep learning approaches have been widely used in structural response analysis [29,30,31]. Previous studies have demonstrated that neural network-based optimization [32,33,34,35] and control strategies [36,37] have a promising potential to improve performance.

Through the literature review, it is seen that most of the previous research studies the pantograph–catenary interaction, which is not relevant to the catenary’s galloping. Only two works [5,28] investigate the aerodynamic instability and preliminarily simulate the galloping behavior. However, the optimization of the catenary’s structural parameters to suppress the galloping has not been undertaken. Thus, it is the motivation of this work to investigate the potential of structural parameters’ optimization in reducing the galloping amplitude of the catenary.

### 1.3. Contribution of This Paper

The prior research underscores the necessity of delving deeper into the aerodynamic instability and galloping of the contact wire, as it is directly related to the operational safety of electrified railway systems subjected to hard wind load. The least economic measure is to make the best use of the potential of the catenary’s structure optimization to suppress the galloping amplitude. This paper attempts to explore the possibility of optimizing the catenary’s structure, including the span length, stagger dropper distribution, and tensions, to reduce the galloping response. Based on a finite element model, the dynamic response of catenary galloping is simulated. The structural parameters of the catenary are changed within a specific prescribed range. The Sobol sequence is implemented to obtain low-discrepancy, quasi-random, and super-uniform distribution sequences for the high-dimensional input of parameters. Then, a surrogate model is built to describe the relationship between the maximum wind deviation of the contact wire and the input parameters. A genetic algorithm is implemented to obtain the optimal parameter settings for suppressing catenary galloping with different constraints.

## 2. Modeling of Catenary Galloping

**Figure 2:**
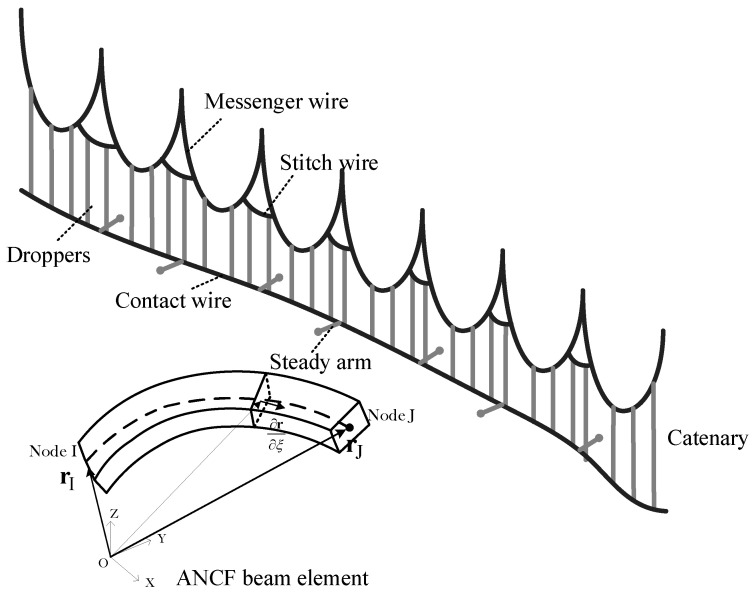
The catenary model with ANCF beam and cable elements.

The catenary system is composed of various intricate components, but when considering its dynamic behaviour subjected to wind load, five key components should be considered. These components are: the contact wire, the messenger wire, the dropper, the stitch wire, and the steady arm, as visually depicted in Figure 2. The primary role of the contact wire is to carry the electrical current intended for collection by the pantograph. The messenger wire and droppers serve the purpose of suspending the contact wire, maintaining it at its designated height. Stitch wires are commonly integrated into certain catenary systems to mitigate elasticity irregularities. To accurately account for the geometric nonlinearity of the messenger, contact, and stitch wires, we employ a nonlinear finite element approach, which has been widely used within various engineering backgrounds [38,39,40,41]. The finite element method is a better representative in describing the flexibility of the catenary when simulating the galloping behaviour than multibody dynamics. For the modeling of the dropper, we utilize an ANCF (Absolute Nodal Coordinate Formulation) cable element, characterized by nonlinear stiffness, in order to account for different operational conditions involving tension and compression. Conversely, the steady arm is represented using a linear truss element, which possesses the capability to pivot around the support point. Additionally, we consider the claws on the clamps as lumped masses within our analysis.

In this research, we have chosen to discretize the contact, messenger, and stitch wires using the ANCF beam element, which provides 12 degrees of freedom (DOFs). For each individual element, the corresponding vector is defined as follows:

(1) $$e={\left[\begin{array}{cccccccccccc} x_{i} & y_{i} & z_{i} & \frac{\partial x_{i}}{\partial \chi } & \frac{\partial y_{i}}{\partial \chi } & \frac{\partial z_{i}}{\partial \chi } & x_{j} & y_{j} & z_{j} & \frac{\partial x_{j}}{\partial \chi } & \frac{\partial y_{j}}{\partial \chi } & \frac{\partial z_{j}}{\partial \chi } \end{array}\right]}^{T}$$

in which *χ* is the local coordinate from 0 to the element length *L*_0_. The derivation of generalized internal elastic force is based on the differentiation of the stain energy, which can be seen as the summation of the contribution from the bending deformation and axial strain deformation. The generalized elastic forces can be written as follows:

(2) $$Q_{e}=\frac{1}{2}\int_{0}^{L_{0}}\left(EA\frac{\partial }{\partial e}\varepsilon_{l}^{2}+EI\frac{\partial }{\partial e}\kappa^{2}\right)d\chi$$

in which *E* is Young’s modulus, *A* is the section area, *I* is the moment inertial of the wire, $\varepsilon_{l}$ is the longitudinal strain defined by Green–Lagrange strain tensor, κ is the curvature of the beam center line, and **r** is the global position vector. The solution of Equation (2) typically results in highly nonlinear elastic forces, which have complex mathematical expressions. However, it is feasible to perform a simplification based on the continuum mechanics assumption [42], which can help to simplify Equation (2) and yield the secant stiffness matrix $K_{e}$ as follows:

(3) $$Q_{e}=K_{e}e$$

The tangent stiffness matrices $K_{T}$ and $K_{L}$ can be obtained by employing the partial of Equation (3) with **e** and *L*_0_ as follows. Here, $K_{L}$ and $\Delta L_{0}$ are only used in the shape-finding procedure.

(4) $$\Delta F=\frac{\partial Q}{\partial e}\Delta e+\frac{\partial Q}{\partial L_{0}}\Delta L_{0}=K_{T}\Delta e+K_{L}\Delta L_{0}$$

A parallel derivation can be applied to obtain the tangent stiffness matrices for the ANCF cable element. It is important to emphasize that when the dropper operates in a slack condition, the axial stiffness effectively reduces to zero. The aggregation of these stiffness matrices from individual elements leads to the formulation of the global incremental equilibrium equation for the entire catenary, as presented below:

(5) $$\Delta F^{G}=K_{T}^{G}\Delta U_{C}+K_{L}^{G}\Delta L_{0}$$

where we denote the global unbalanced force vector as $\Delta F^{G}$, while $K_{T}^{G}$ and $K_{L}^{G}$ represent the global stiffness matrices corresponding to the incremental nodal displacement vector $\Delta U_{C}$ and the incremental unstrained length vector $\Delta L_{0}$, respectively. To meet the specific design requirements of the catenary [43], we introduce additional constraint conditions aimed at constraining undesired movements. Upon establishing the initial configuration of the catenary, the global stiffness matrix $K_{T}^{G}$ can be assembled to reflect the equilibrium state of the catenary. This matrix dynamically changes as the catenary deforms in response to wind loads. When coupled with a coherent mass matrix $M_{T}^{G}$ and damping matrix $C_{T}^{G}$, the equation of motion for the catenary, subjected to wind-induced excitation represented by the vector $F_{T}^{G}\left(t\right)$, can be expressed as follows:

(6) $$M_{T}^{G}{\ddot{U}}_{C}\left(t\right)+C_{T}^{G}{\dot{U}}_{C}\left(t\right)+K_{T}^{G}\left(t\right)U_{C}\left(t\right)=F_{T}^{G}\left(t\right)$$

Equation (6) can be solved by a time-integration method to obtain the dynamic response of the catenary at each time instant *t*.

**Figure 3:**
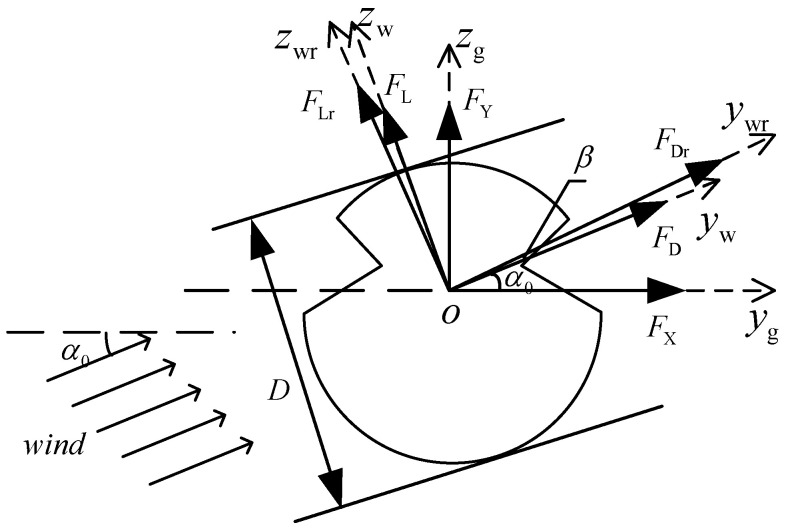
Sketch of contact-wire cross-section with wind load.

As illustrated in Figure 3, the aerodynamic forces acting on the cross-section of the contact wire can be categorized into two components: lift ($F_{L}$) and drag ($F_{D}$). Figure 3 provides a visual representation of a contact wire cross-section exposed to a wind load represented by *U* with an angle of attack $\alpha_{0}$. The drag force $F_{D}$ aligns itself with the direction of the wind, while the lift force $F_{L}$ acts perpendicular to the wind direction *U*. To derive the aerodynamic forces exerted on the contact wire, we establish three coordinate systems. The first one, denoted $y_{w}-o-z_{w}$, is determined by the initial angle of attack $\alpha_{0}$. The second, labeled $y_{wr}-o-z_{wr}$, is determined by the effective angle of attack $\alpha_{r}=\alpha_{0}+\beta$, which accounts for the dynamic wind angle resulting from the contact wire’s motion within the fluid. The third coordinate system, designated $y_{g}-o-z_{g}$, is aligned with the global reference framework of the catenary model. In the $y_{wr}-o-z_{wr}$ coordinate system, the lift $F_{L}$ and drag $F_{D}$ can be mathematically expressed as follows, as derived from reference [4]:

(7a) $$F_{\mathrm{Dr}}=0.5\rho_{\mathrm{air}}U_{r}^{2}DLC_{D}(\alpha_{r})$$

(7b) $$F_{\mathrm{Lr}}=0.5\rho_{\mathrm{air}}U_{r}^{2}DLC_{L}(\alpha_{r})$$

in which $C_{D}(\alpha_{r})$ and $C_{L}(\alpha_{r})$ represent the drag and lift coefficients at the actual angle of attack $a_{r}$. The relative wind velocity $U_{r}$ is defined as follows:

(8) $$U_{r}=\sqrt{{\left(U-{\dot{x}}_{p}\right)}^{2}+{\left({\dot{x}}_{h}\right)}^{2}}$$

in which ${\dot{x}}_{p}$ and ${\dot{x}}_{h}$ are the horizontal and vertical velocities of the contact wire, respectively. The dynamic wind angle β induced by the movement of the contact wire within the fluid can be expressed by

(9) $$\beta =-\arctan({\dot{x}}_{h}/(U-{\dot{x}}_{p}))$$

In $y_{w}-o-z_{w}$, the drag $F_{D}$ and lift $F_{L}$ can be expressed by

(10a) $$F_{D}=F_{\mathrm{Dr}}\cos\left(\beta \right)-F_{\mathrm{Lr}}\sin\left(\beta \right)$$

(10b) $$F_{L}=F_{\mathrm{Dr}}\sin\left(\beta \right)+F_{\mathrm{Lr}}\cos\left(\beta \right)$$

Then, the aerodynamic forces used in the finite element model are achieved through a simple coordinate transformation.

## 3. Preliminary Analysis of Catenary Galloping Response

**Figure 4:**
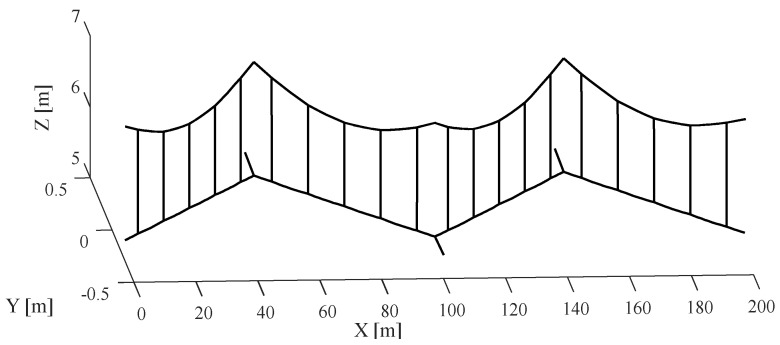
Initial configuration of central fours spans of the OCL.

**Figure 5:**
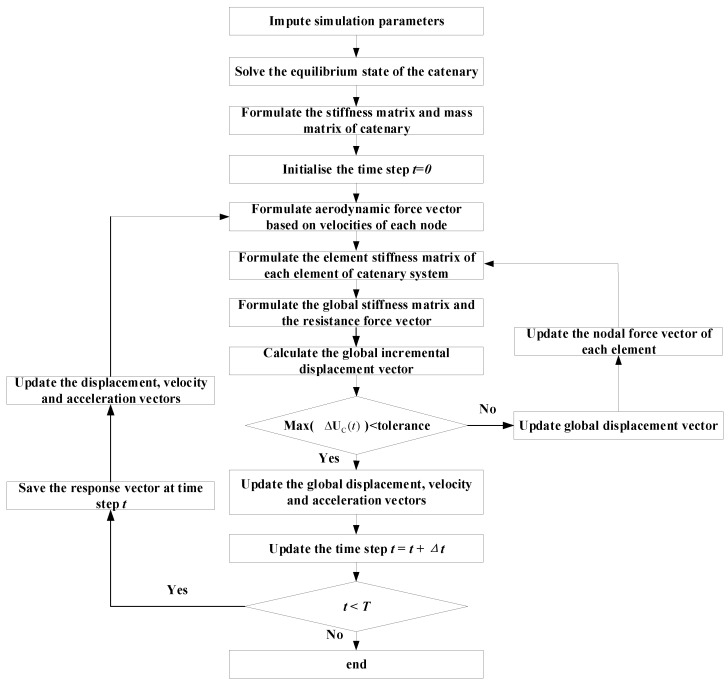
Flow chart for simulating catenary galloping response.

**Figure 6:**
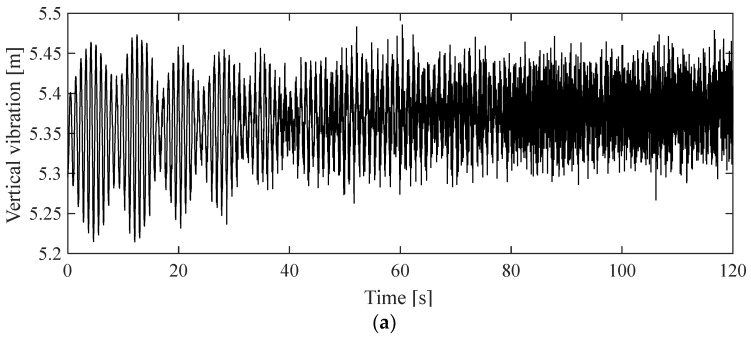

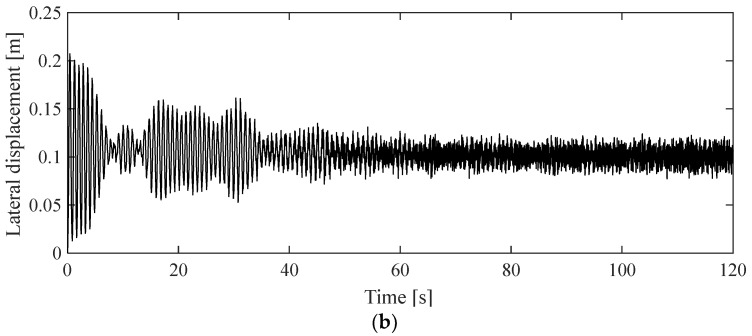
Time history of contact-wire displacement at the mid-span point in vertical (**a**) and lateral (**b**) displacement.

To accurately simulate galloping, a crucial initial step involves selecting appropriate aerodynamic coefficients that align with the conditions conducive to galloping. Based on insights gleaned from wind tunnel experiments, it becomes evident that aerodynamic instability is likely to occur at a significant wear level and specific angles of attack. In alignment with findings from [5], this study opts for a 9° angle of attack and a 20% wear of the contact wire. These defined parameters serve as the foundation for the subsequent numerical simulation of catenary galloping, aiming to unravel the impact of catenary geometry on the galloping response. The construction of a 10-span catenary model, representative of a typical conventional railway, employs the parameters outlined in Table 1. Figure 4 visually encapsulates the initial configuration of the central four spans of the catenary. The simulation procedure adopted in Figure 5 is used to implement the simulation of the catenary galloping. A self-programmed code package is used in the simulation, which has been developed by the authors’ research group over several years [44]. The analytical framework adopts the track reference frame, with the contact wire positioned at a height of 5.3 m relative to the track surface. The pivotal mid-span point of the contact wire emerges as the primary focus for analysis, providing a quantitative measure of the deviation from its initial configuration. The ensuing investigation unfolds through the lens of vertical and lateral displacement, as depicted in Figure 6a,b, respectively. These results illuminate the dynamic interplay between catenary geometry parameters and the ensuing galloping response, thereby contributing valuable insights to the understanding of this intricate phenomenon.

The findings indicate that, following a 60 s simulation, the galloping response stabilizes. Notably, the vertical vibration exhibits greater intensity compared to its lateral counterpart. In the vertical direction, the maximum amplitude surpasses 0.2 m, while the maximum lateral amplitude peaks at approximately 0.05 m. The most substantial deviation observed in the contact-wire point from its initial position amounts to 0.248 m. This particular scenario, assessed with the original parameters, serves as a benchmark for evaluating the effectiveness of the optimization strategy. It provides a foundational reference point against which the outcomes of the optimization approach can be measured.

## 4. Effect of Geometry Parameters on Catenary Galloping Response

Given the intricate nature of the catenary structure, it encompasses various structural parameters such as span length, tension in both messenger and contact wires, dropper interval, dropper number, and stagger value. The simulation of catenary galloping, characterized by significant nonlinearity, raises concerns about simulation efficiency when exploring the impact of these structural parameters on the galloping response. The tremendous computational effort in the finite element simulation makes it impossible to connect the finite element model with the algorithm directly. A surrogate model has to be developed to replace the previous finite element model. In our simulation, each case takes about 6 h to simulate a 60 s galloping response. In the optimization, the algorithm needs to run over ten thousand cases, which is almost an unachieved target. Therefore, the implementation of an efficient sampling method becomes imperative to ensure a more comprehensive coverage of the high-dimensional sample space. In this study, we employ a well-established Sobol sequence to extract samples from specific ranges of structural parameters associated with the catenary. Six distinct types of structural parameters are considered, namely messenger-wire tension, contact-wire tension, span length, stagger value, dropper-to-steady-arm interval, and dropper number. Each variable is subject to constraints defined in accordance with the design specifications:

10 kN < Messenger-wire tension < 20 kN

10 kN < Contact-wire tension < 20 kN

45 < Span length < 60 m

0.1 < Stagger value < 0.3 m

4 < Dropper number < 8

4.5 m ≤ Steady-arm-to-dropper interval ≤ 7 m

### 4.1. Sobol Sequence

The biggest challenge in optimizing the catenary structure for reducing the galloping amplitude is the considerate computational effort. It is almost impossible to connect the finite element model with the optimization algorithm directly. We have to rely on the neural network that serves as a surrogate model to replace the finite element model in the optimization. The neural network should be trained with a good dataset that can represent the overall characteristics of the finite element model. Here, the Sobol sequence is a deterministic, low-discrepancy sequence of points used in numerical simulations, particularly in quasi-Monte Carlo methods for high-dimensional integration and sampling problems. It is designed to provide a more-even coverage of the sample space compared to purely random sequences, making it especially useful for reducing variance in Monte Carlo simulations. The Sobol sequence is constructed using a set of primitive polynomials and bit-wise operations. The key idea is to generate points that cover the sample space systematically, ensuring that each dimension is equally represented. The points are typically generated in the unit hypercube ([0, 1]^d^), where ‘d’ is the dimensionality of the problem. The Sobol sequence is defined recursively using the following equations:

For each dimension *i*, we initialized the Sobol sequence with:

(11) $$X_{i}\left(0\right)=0.5$$

For each dimension *i* and for each term *j*, we computed the Sobol point as follows:

(12) $$X_{i}\left(j\right)=X_{i}\left(j-1\right)⊕\left(V_{j}<<\left(1-c_{j}\right)\right)$$

Here:

$X_{i}\left(j\right)$ is the Sobol point for dimension *i* at term *j* [45]. ⊕ denotes bitwise XOR (exclusive or), which is a logical operator and has a negation that corresponds to the logical biconditional. In the case of two inputs, XOR evaluates to true only when the inputs differ (one is true, and the other is false). When dealing with multiple inputs, XOR is true if and only if the count of true inputs is odd [46]. $V_{j}$ is a direction vector obtained from primitive polynomials. c(j) is the position of the rightmost zero bit in *j*.

**Figure 7:**
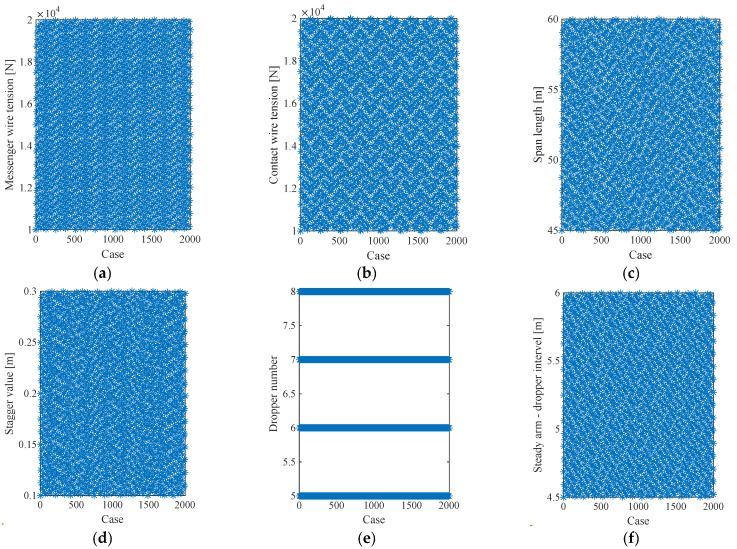
Sobol sequences for each parameter. (**a**) messenger-wire tension, (**b**) contact-wire tension, (**c**) span length, (**d**) stagger value, (**e**) dropper number and (**f**) steady arm–dropper interval.

The key to Sobol sequences is the use of these direction vectors and bitwise operations to ensure that the points are well-distributed across the hypercube. Therefore, Sobol sequences are highly effective in reducing the variance of Monte Carlo simulations, especially for high-dimensional problems, compared to random sequences like pseudo-random numbers. They are widely used in applications such as finance, physics, and computer graphics, where accurate numerical integration and sampling are essential. Using the Sobol sequence method with a sampling number of 2000, the samplings of the structural parameter are presented in Figure 7. It is seen that with the help of the Sobol sequence, each parameter is evenly distributed within the specific range.

### 4.2. Simulation Results with Random Parameters

**Figure 8:**
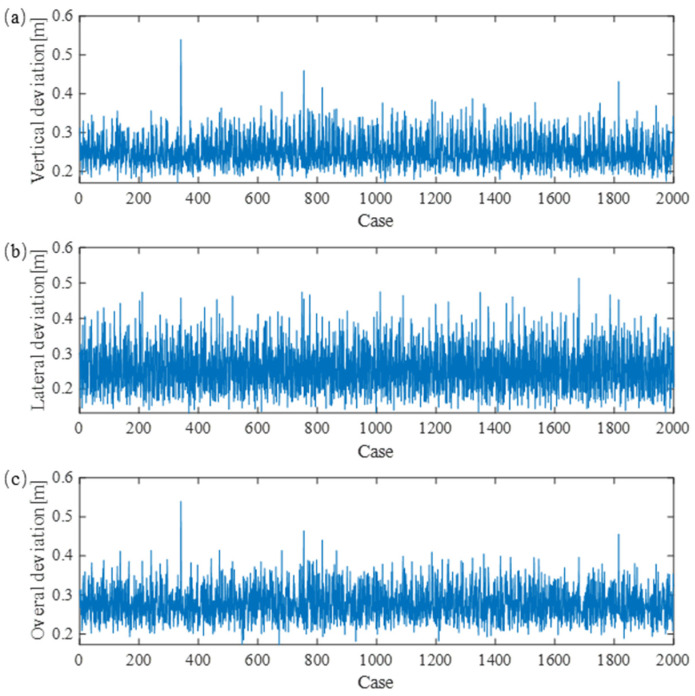
Maximum deviation of the mid-span point on the contact wire. (**a**) presents the results in vertical direction, (**b**) presents the results in lateral direction, (**c**) presents the results of overall deviation.

In the numerical simulation, the damping ratio is assumed to be constant as the measurement data collected in [47] proves that the change of structural parameters does not significantly affect the damping ratio. Through 2000 numerical simulations, the resulting galloping responses can be obtained. The displacement on the mid-span point is adopted as the analysis object. The maximum deviations with respect to the initial position are adopted as the main indices to evaluate the intensity of the galloping. The maximum vertical, lateral and the overall deviations of the mid-span point on the contact wire evaluated with 2000 cases are presented in Figure 8a–c, respectively. It is seen that the structural parameters’ effect on the galloping response is noticeable. The overall deviation ranges from 0.2 m to 0.5 m. The worst performance appears in case 341, while the best one appears in case 1126. The structural parameters for the worst and best cases are presented as follows:

- Worst Case (Case 341):

Messenger-wire tension: 11,660.16 N; Contact-wire tension: 10,097.66 N;

Span length: 58.51 m; Stagger value: 0.1762 m;

Dropper number: 6; Steady arm–dropper interval: 5.97.

- Best Case (Case 1126):

Messenger-wire tension: 19,736.33 N; Contact-wire tension: 16,552.73 N;

Span length: 45.19 m; Stagger value: 0.2299 m;

Dropper number: 5; Steady arm–dropper interval: 5.54.

The aforementioned parameters reveal a notable trend: a shorter span length and higher tension are generally advisable for mitigating galloping. This observation aligns with our intuitive understanding that a smaller span length and increased rigidity enhance wind-resistance capabilities. While these factors contribute significantly, there remains potential for mitigating the adverse effects of galloping through adjustments in other parameters. In the upcoming section, we delve into this untapped potential through the application of an optimization method, seeking to further explore avenues for enhancing the system’s resilience against galloping.

## 5. Optimization Based on Neural Networks

**Figure 9:**
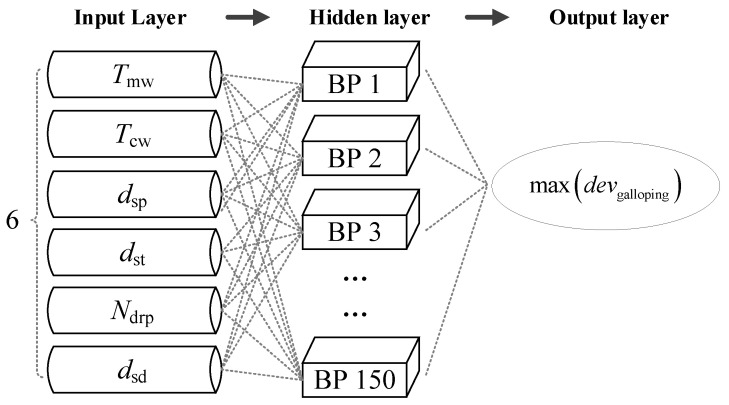
Neural network architecture.

In this study, a neural network optimization algorithm designed for tackling nonlinear least-squares problems is employed to optimize the parameters of the catenary, with the aim of minimizing the contact-wire deviation induced by galloping. The optimization process unfolds in two key steps. Firstly, a surrogate model for catenary galloping is established through an artificial neural network, utilizing the database acquired in the preceding section. Subsequently, a genetic algorithm, as outlined by [48], is integrated into the optimization framework. Here, the genetic algorithm can be replaced by any classic optimization approach, such as particle swarm [49]. The relationship that is expected to be established is between the maximum galloping displacement (which is an index) and the main structural parameters of the catenary. The target of this work is not very challenging as the galloping time history is not taken as the prediction object, as reported in [50]. This genetic algorithm is geared towards minimizing the sum of squares of a vector-valued objective function. To accomplish this, a standard Back-Propagation Neural Network (BPNN) is employed—a multi-layer feed-forward network comprising multiple neurons. The BPNN algorithm iteratively minimizes the error between the network’s output and the expected output by adjusting the weights and thresholds. The choice of a BPNN is motivated by its demonstrated superiority in accuracy and generalization capabilities compared to traditional multivariate regression functions used in Response Surface Methodology (RSM). In our investigation, the Bayesian regularization backpropagation serves as the training function for the neural network. This function leverages the Levenberg–Marquardt optimization method to update the weight and bias values of the network [51]. Throughout the training process, these weights and biases are adjusted to minimize the network error. The network’s performance is evaluated using the mean square error (MSE), with a predefined target value set at 4 × 10^−7^ for MSE. The maximum number of training epochs is capped at 100, and the learning rate is established at 0.1. The neural network architecture, as depicted in Figure 9, features 150 BP neurons in the hidden layer. The selection of the number of neurons in the hidden layer is contingent upon the problem’s complexity; insufficient nodes may compromise fitting ability, while an excess may lead to overfitting. In this instance, a deliberate choice of 150 BP neurons is made to strike a balance between model complexity and effectiveness.

The neural network architecture is presented in Figure 9. The neural network serves as a virtual internal objective function, equivalent to the objective function defined to reduce the maximum wind deviation, while the genetic algorithm is employed to minimize the output of the neural network. It is noteworthy that the optimum solution obtained by the genetic algorithm for the neural network is tantamount to the optimum solution for the objective function, given the equivalence between the neural network and the objective function. Diverging from conventional optimization procedures [52], the simulation data aren’t directly utilized as inputs for the genetic algorithm. Instead, an initial set of training data is simulated for the purpose of training the neural network. Subsequently, the trained neural network functions as the objective function in the genetic algorithm optimization process. The optimal solution identified by the genetic algorithm is cross-verified with numerical simulation data. If convergence criteria are not met, additional data are simulated to further train the neural network. This neural network optimization approach has proven to be more efficient in yielding optimal results compared to traditional genetic algorithms, as demonstrated in previous studies [53].

Normally, the span length and the contact-wire tension for a catenary are determined before the optimization is performed. The former depends on the rail network’s commercial demand, while the latter is determined by the limitations of wave speed and material property [54]. Here, we present two cases, as follows, based on practical engineering experience. To save the economic cost of the construction, a long span length is normally preferred [1]. Therefore, the span length and contact-wire tension are restricted according to the design data, as follows:

Contact-wire tension: 15 kN; Span length: 50 m.

With the above constraints, other parameters, including the messenger wire, stagger value, dropper number, and dropper–steady arm interval are taken as the variables that we sought to optimize. The objective function is to minimize the maximum deviation of the contact wire against its initial position. Thus, the optimization problem reads:

Objective: $\min\;\max\left(dev_{c}\left(D_{\mathrm{sd}},N_{\mathrm{dd}},D_{\mathrm{stag}},T_{\mathrm{mw}}\right)\right)$

Design constraints: *T*_cw_ = 15,000 N; *L*_s_ = 50 m.

**Figure 10:**
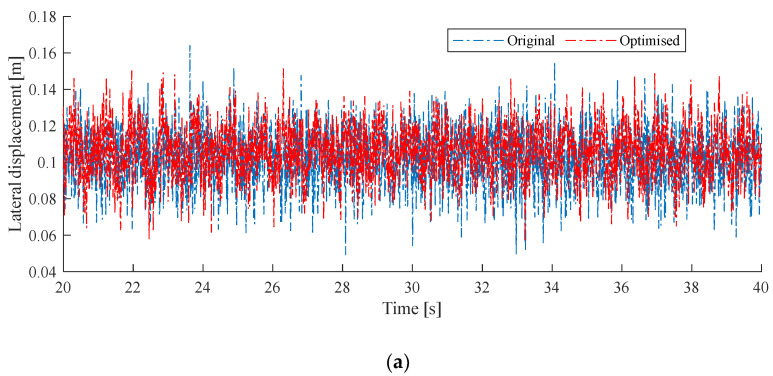

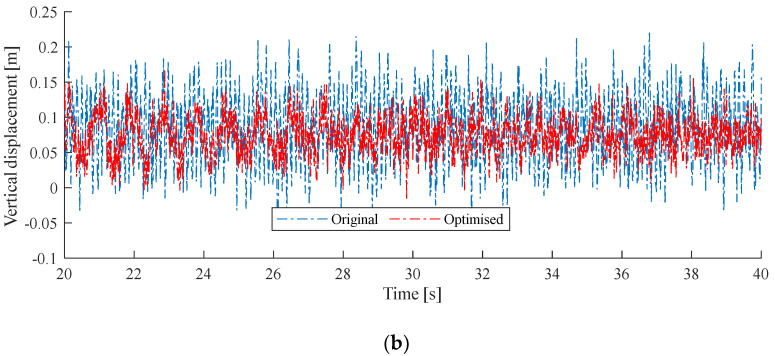
Comparison of galloping behaviors between the original and optimized results for case 1. (**a**) presents the lateral displacement; (**b**) presents the vertical displacement.

Then, the genetic algorithm is implemented to solve the optimization problem. Figure 10 shows the comparison between the original and optimized results. The optimized parameters are $T_{\mathrm{mw}}=20,000\;N$, $D_{\mathrm{stag}}=0.144\;m$, $N_{\mathrm{dd}}=5$ and $D_{\mathrm{sd}}=4.499\;m$. The maximum deviation of the contact-wire mid-span point with respect to its initial position is taken as the analysis index. It is seen that the reduction in vertical displacement is more significant than the lateral displacement. In the lateral direction, the maximum deviation is reduced from 0.164 m to 0.151 m—7.93%. In the vertical direction, the maximum deviation is reduced from 0.216 m to 0.167 m—22.69%. As the increase in the messenger-wire tension always has a positive effect in suppressing the galloping response, the optimized messenger-wire tension reaches the upper bound of the specified range. In order to explore the potential of adjusting other parameters in reducing the galloping response, the messenger-wire tension is also restricted in the following case.

Contact-wire tension: 15 kN; Messenger-wire tension: 15 kN; Span length: 50 m;

With the above constraints, the optimization problem reads:

Objective: $\min\;\max\left(dev_{c}\left(D_{\mathrm{sd}},N_{\mathrm{dd}},D_{\mathrm{stag}}\right)\right)$

Design constraints: *T*_cw_ = 15,000 N; *L*_s_ = 50 m; *T*_mw_ = 15,000 N.

**Figure 11:**
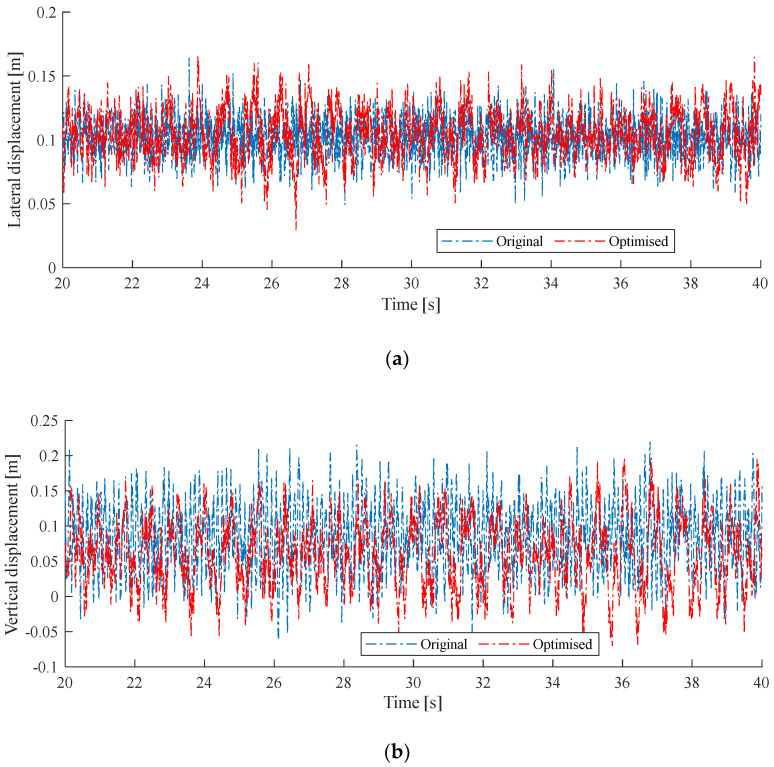
Comparison of galloping behaviors between the original and optimized results for case 2. (**a**) presents the lateral displacement; (**b**) presents the vertical displacement.

Then the genetic algorithm is implemented to solve the optimization problem. Figure 11 shows the comparison between the original and optimized results. The optimized parameters are $D_{\mathrm{stag}}=0.196\;m$, $N_{\mathrm{dd}}=5$ and $D_{\mathrm{sd}}=4.499\;m$. It is seen that even though the messenger-wire and contact-wire tensions are restricted, the adjustment of stagger value, dropper number, and dropper–steady arm distance still has the potential to reduce wind deviation. There is no significant reduction in the maximum lateral deviation, while the maximum vertical deviation is reduced from 0.216 m to 0.195 m—9.72%. The optimized dropper number and the steady arm–dropper distance are the same as in the previous case. It is concluded that a shorter steady arm–dropper distance is preferred for reducing the galloping response.

It is demonstrated from the above analyses that the most important parameters affecting the galloping response are the span length and the tension level. In particular, a reduction in the span length and an increase in the contact-wire tension can significantly decrease the galloping amplitude. The two case studies demonstrate that the messenger-wire tension also has significant effects on suppressing galloping. However, the other two parameters (stagger and dropper distribution) have very limited effect in reducing the galloping amplitude, as demonstrated in the second case study. However, the increase in tension will aggravate the stress in the messenger and contact wires. The reduction in span length will boost the construction cost. That is why, in the case study, we constrain the contact-wire tension and span length to avoid the extra cost caused by the optimal parameter settings. Therefore, the optimization results in the two case studies cannot significantly increase the cost. Regarding the difficulties in operability, we agree that the optimal results are a bit academic and are difficult to accurately achieve in reality due to the construction tolerance. Specifically, it is difficult to achieve an accurate dropper distribution. The temperature variation can cause a geometry deviation, as reported in [21]. Therefore, it is difficult to maintain a constant dropper distribution in reality. Apart from structural optimization, some other measures may be considered for galloping suppression. Firstly, optimizing the contact-wire profile emerges as a promising approach. The alteration of the contact-wire cross-section profile stands out as the primary factor contributing to the catenary’s galloping. Through the optimization of this profile, we anticipate mitigating or even preventing the occurrence of catenary galloping. Secondly, introducing damping components represents another viable strategy. The galloping of the catenary is essentially a self-excited vibration triggered by negative damping. Incorporating damping components into the system can counteract this negative damping, thereby enhancing the stability of the catenary system.

## 6. Conclusions

This paper investigates the potential for optimizing the structure of the catenary system to mitigate galloping responses. The optimization parameters include span length, stagger dropper distribution, and tension levels. Utilizing a finite element model, the dynamic response of catenary galloping is simulated, and structural parameters are varied within defined ranges. To generate low-discrepancy, quasi-random, and super-uniform-distribution sequences for high-dimensional parameter inputs, the Sobol sequence is employed.

Subsequently, a genetic algorithm based on neural networks is applied to identify optimal parameter settings for suppressing catenary galloping, considering various constraints. The findings suggest that adjusting structural parameters can indeed reduce the galloping amplitude of the railway catenary. The most effective approach involves increasing tension and decreasing span length. Even with a fixed tension and span length, adjusting other parameters proves effective in reducing galloping amplitudes. Specifically, maintaining a moderate stagger value and a short steady arm–dropper distance is recommended for achieving the minimum galloping amplitude.

## Figures and Tables

**Table 1:** Main parameters of OCL.

**OCL Geometrical Property**	
Encumbrance: 1.6 m; Interval of droppers: 10 m; Span: 50 m; Number of droppers: 5	
**Material property**	
Contact line	Line density: 1.082 kg/m; Cross-section area: 120 mm^2^; Tensile rigidity: 10^6^ N/m; Tension: 15 kN
Messenger line	Line density: 1.068 kg/m; Cross-section area: 120 mm^2^; Tensile rigidity: 10^6^ N/m; Tension: 15 kN
Dropper	Line density: 0.14 kg/m; Tensile rigidity: 10^5^ N/m

## Data Availability

Data are contained within the article.
